# Supplementary material for: Prevalence of hepatitis B and C virus infections in Lao People’s Democratic Republic: The first national population-based cross-sectional survey
Source: PLoS One. 2022 Dec 30;17(12):e0278933. doi: 10.1371/journal.pone.0278933 (PMC9803141; doi:10.1371/journal.pone.0278933)
Supplement: Attachment — Submitted filename: Response to Reviewers.docx [file pone.0278933.s005.docx]

DATE: October 28, 2022

Emily Chenette

Editor-in-Chief

*Plos One*

Re: Manuscript ID: PONE-D-22-13251R1

Dear Editor,

We appreciate your e-mail and review of the manuscript (PONE-D-22-13251R1) that we sent on August 26, 2022. We thank one reviewer for providing a suggestion regarding the improvement of the manuscript.

All changes have been made in response to the reviewers’ suggestion, and itemized response to the individual reviewer’s comment is also attached as below.

**Reviewer #1:**

Dear authors,

The manuscript was improved considerably. Most of my queries were addressed. However, I would suggest that a careful review of the language be carried out before publication.

We appreciate the reviewer's suggestion. We requested English proofreading by native English speakers and made some corrections and revisions.

We believe that we have responded as the reviewer suggested and hope that the revised manuscript is now acceptable for publication in PLOS ONE. Thank you for your generous consideration.

Sincerely,

Shinsuke Miyano

Bureau of International Health Cooperation, National Center for Global Health and Medicine (NCGM)

Postal Address: 1-21-1, Toyama, Shinjuku-ku, Tokyo, 1628655, Japan

Phone No: +81-(0)3-3202-7181 (ext. 2720)

Fax No: +81-(0)3-3205-7860

Email Address: [s-miyano@it.ncgm.go.jp](mailto:s-miyano@it.ncgm.go.jp)
